# Synthesis and Characterization of Zeolite NaY Dispersed on Bamboo Wood

**DOI:** 10.3390/ma16144946

**Published:** 2023-07-11

**Authors:** Pimrapus Tawachkultanadilok, Nattawut Osakoo, Chalermpan Keawkumay, Krittanun Deekamwong, Narongrit Sosa, Catleya Rojviriya, Supinya Nijpanich, Narong Chanlek, Sanchai Prayoonpokarach, Jatuporn Wittayakun

**Affiliations:** 1 School of Chemistry, Institute of Science, Suranaree University of Technology, Nakhon Ratchasima 30000, Thailand; 2 Institute of Research and Development, Suranaree University of Technology, Nakhon Ratchasima 30000, Thailand; 3 Functional Materials and Nanotechnology Center of Excellence, School of Science, Walailak University, Nakhon Si Thammarat 80160, Thailand; 4 Synchrotron Light Research Institute, Nakhon Ratchasima 30000, Thailand

**Keywords:** zeolite NaY, bamboo wood, zeolite–wood composite, zeolite particle dispersion, nickel adsorption

## Abstract

Zeolites in powder form have the potential to agglomerate, lowering access to active sites. Furthermore, a suspension of fine zeolite powder in liquid media is difficult to separate. Such drawbacks could be improved by dispersing zeolite crystals on support materials. This work demonstrates the dispersion of zeolite NaY crystals on bamboo wood by mixing the wood with zeolite gel before hydrothermal treatment. The syntheses were performed with acid-refluxed and non–refluxed wood. The phase of zeolites, particle distribution and morphology, zeolite content in the wood, and zeolite–wood interaction were investigated using X-ray diffraction, X-ray tomography, scanning electron microscopy, thermogravimetric analysis, nitrogen sorption analysis, and X-ray photoelectron spectroscopy. Higher zeolite content and better particle dispersion were obtained in the synthesis with the acid–refluxed wood. The composite of NaY on the acid-refluxed wood was demonstrated to be an effective adsorbent for Ni(II) ions in aqueous solutions, providing a higher adsorbed amount of Ni(II) per weight of NaY.

## 1. Introduction

Zeolites have been widely utilized in research and industry as catalysts [1], gas separations [2], and adsorbents [3]. Employing fine zeolite powder in a liquid medium is typically problematic due to the tendency of particle agglomeration and difficulty separating particles from the media. One approach to solve these issues is to disperse zeolite on a support material.

Zeolite can be dispersed to form composites with various substrates (Table 1), including polymers [4], ceramics [5], carbon from various sources [6,7,8,9], cellulose [10,11,12], and wood [13]. Valtchev et al. [11] prepared wood–zeolite composites between zeolite A (LTA) and wood substrates with different amounts of lignin and cellulose. They suggested that hydroxide ions from the aluminosilicate gel, generating hydroxyl groups on cellulose, facilitate the zeolite nucleation. However, lignin from the biomass substrate could limit the crystallization of zeolite A [12]. Chemical treatment is, therefore, necessary to eliminate the lignin and enhance the amount of cellulose. Recently, Krukkratoke et al. [13] reported the synthesis of zeolite NaY (FAU) in lead tree wood through a hydrothermal method. Zeolite NaY was the major phase in the acid–refluxed wood, whereas zeolite NaP (GIS) was produced in the non–refluxed wood. The composition and morphology could influence the formation of zeolite. In addition, wood could absorb water from the zeolite gel composition, leading to higher alkalinity and accelerating the crystallization process. 

Bamboo wood is of interest in this work. It is abundant in tropical and subtropical countries. The cellular structure of bamboo has large macropores ranging from around 100 μm in vessels, between 10 and 50 μm in phloems, to about 5 μm triangular pores [14]. There are no reports on using bamboo wood as a substrate for zeolite deposition.

This work aims to prepare composites consisting of zeolite NaY dispersed on acid-refluxed and non–refluxed bamboo wood. The composites are investigated using X-ray diffraction (XRD), X-ray tomographic microscopy (XTM), scanning electron microscopy (SEM), thermogravimetric analysis (TGA), N_2_ adsorption–desorption analysis, and X-ray photoelectron spectroscopy (XPS) to determine the composite phases, porosity, zeolite distribution, and interactions between the zeolite and bamboo wood. Finally, the wood–zeolite composite is utilized as an adsorbent to remove nickel ions from aqueous solutions. 

## 2. Materials and Methods

### 2.1. Materials and Reagents

Bamboo wood was collected from Suranaree University of Technology (SUT), Nakhon Ratchasima, Thailand. Sodium hydroxide (NaOH, 97% wt.) and hydrochloric acid (HCl, 37%) were obtained from Carlo Erba (Val de Reuil, France) and ANaPURE (Auckland, New Zealand), respectively. Fumed silica (SiO_2_, 99.8%) and sodium aluminate anhydrous (NaAlO_2_ anhydrous: Al_2_O_3_, 55–56%) were purchased from Sigma-Aldrich (Steinheim, Germany). Na_2_SiO_3_ solution was prepared by dissolving 28.7 g of fumed silica in 11.48 g of NaOH and 59.82 g of deionized (DI) water. Nickel ion (Ni(II)) solution (100 ppm) was prepared by dissolving Ni(NO_3_)_2_·6H_2_O (RPE grade, Carlo Erba (Val de Reuil, France)) in DI water.

### 2.2. Pretreatment of Bamboo Wood

Bamboo culms were peeled and the wood was cut into small cylindrical pellets with dimensions of 5 mm × 5 mm (diameter × height). The wood pellets were ultrasonically cleaned with DI water and dried at 90 °C for 24 h to obtain bare wood (W). Subsequently, the pellets were treated with 67 mL of 3 M HCl solution at 90 °C for 6 h, separated, washed, and dried at 90 °C overnight to obtain acid-refluxed wood (RW).

### 2.3. Synthesis of Wood–Zeolite Composite

**Scheme 1:**
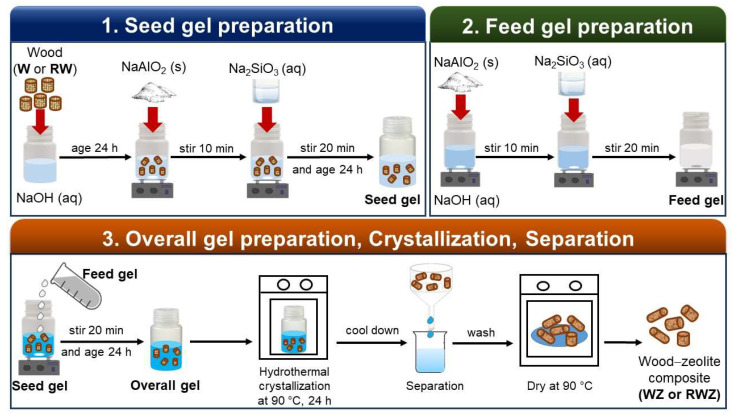
The synthesis of wood–zeolite composites including 1. seed gel preparation, 2. feed gel preparation, and 3. overall gel preparation, crystallization, and separation.

The wood–zeolite composites were synthesized by adding a feed gel to a mixture of seed gel of zeolite NaY containing pellets of W or RW. The synthesis steps are summarized in Scheme 1. The composition of the seed and feed gels were modified from the literature [15]. The mixture of seed gel and wood was prepared by immersing 1.00 g of wood (W or RW) in a solution consisting of 1.00 g of NaOH and 10.00 g of DI water for 24 h and adding 0.418 g of anhydrous NaAlO_2_ under magnetic stirring. Then, 4.79 g of Na_2_SiO_3_ solution was slowly dropped (40 drops/min) into the mixture. The mixture was stirred for 30 min and aged for 24 h.

To prepare the feed gel, 2.78 g of NaAlO_2_ was dissolved in a solution containing 0.035 g of NaOH and 22.3 g of DI water. Next, 30.19 g of Na_2_SiO_3_ silicate solution was slowly added to the sodium aluminate solution at a rate of 60 drops/min under stirring for 30 min. The obtained feed gel was then added to the wood-containing seed gel and stirred continuously for 30 min. The resultant mixture was aged for 24 h at room temperature without stirring, then heated at 90 °C for 24 h. After cooling down, the solid products, including the wood–zeolite composite and zeolite powder, were separated, repeatedly washed with DI water using sonication until the pH of the washing water was approximately 8, and dried at 100 °C overnight. The obtained pellets were named untreated wood–zeolite composite (WZ) and refluxed wood–zeolite composite (RWZ).

### 2.4. Characterization Methods

Phases of bamboo wood and the wood–zeolite composite were studied using X-ray diffraction (XRD, Bruker D8 ADVANCE, Bruker AXS GmbH, Karlsruhe, Baden-Württemberg, Germany) with Cu Kα radiation (λ = 1.5406 Å) operated at 40 kV of voltage and 30 mA of current with a step size of 0.02° and speed of 0.4 s/step. The three-dimensional (3D) structure of samples was investigated using the synchrotron radiation X-ray tomographic microscopy (XTM) technique at Beamline 1.2W, Siam Photon Source Facility, Synchrotron Light Research Institute (SLRI). X-ray projections of the samples were collected for 180 degrees with 0.1 angular increments to form a dataset. Polychromatic X-rays were attenuated with 200 micron-thick aluminum foil, to obtain a mean energy of 10 keV, in order to minimize the artifacts. The X-ray projections were acquired with a pixel size of 1.44 μm by using an sCMOS PCO.edge 5.5 camera Excelitas PCO GmbH, Kelheim, Germany). The tomographic data were pre-processed and calculated for reconstructed slices based on a filtered-back projection algorithm using Octopus Reconstruction software (Version 8.9.4, TESCAN XRE, Ghent, Belgium). To identify and classify the distribution of zeolite on untreated and refluxed woods and the porosity of both wood–zeolite composites, Octopus Analysis software (Version 1.1.4, TESCAN XRE, Ghent, Belgium) was used to compare each voxel to all the surrounding voxels. Finally, a 3D reconstructed image of the tomographic volumes of the composite was visualized using Drishti software Version 2.6.4 [16,17]. 

The morphology of the samples was obtained using a field emission scanning electron microscope (FE-SEM, JEOL JSM 7800F, JEOL Ltd., Akishima, Tokyo, Japan). The wood pellet was spread on a layer of silver paint adhered to a metal stub and coated with gold under an argon atmosphere. Elemental analysis was performed using energy-dispersive X-ray spectroscopy (EDS) (Carl Zeiss NTS GmbH, Oberkochen, Baden-Württemberg, Germany). The surface elements of the wood–zeolite composites were analyzed using X-ray photoelectron spectroscopy (XPS) on a PHI 5000 *VersaProbe* II XPS system (ULVAC-PHI, Kanagawa, Japan) with Al Kα radiation as the excitation source. The binding energies were calibrated using the C 1s peak (284.8 eV). The functional groups of the obtained samples were analyzed using Fourier transform infrared spectroscopy (FTIR) in attenuated total reflectance (ATR) mode. The measurements were conducted on a Bruker Tensor 27 FTIR instrument (Bruker Corporation, Billerica, MA, USA) with a resolution of 4 cm^−1^ and 128 scans for each sample.

The thermal degradation of the wood and wood–zeolite composite was determined through thermogravimetric analysis (TGA, Mettler Toledo model TGA/DSC1, Mettler-Toledo AG Analytical, Zürich, Schwerzenbach, Switzerland). About 5–20 mg of each sample in an alumina pan was heated to 800 °C at 10 °C/min under an airflow of 50 mL/min. The textural property of the samples was determined through N_2_ sorption analysis at −196 °C on a Belsorp mini II instrument (BEL Japan, Inc., Osaka, Toyonaka-shi, Japan). All samples were degassed at 70 °C under a vacuum for 24 h. The surface area was calculated according to the Brunauer–Emmett–Teller (BET) method. 

### 2.5. Adsorption Experiment

The adsorption efficiency of the wood–zeolite composites was evaluated in Ni(II) solutions. To compare the adsorption capacity of the wood–zeolite composite, zeolite NaY was synthesized in similar conditions without wood addition. The zeolite pellet (diameter = 5 mm) was prepared by pressing the powder with 1-ton hydraulic pressure. In the adsorption study, 20 mL of 100 ppm Ni(II) solution with one pellet of an adsorbent was stirred for 3 h at room temperature in a polypropylene bottle. Then, the adsorbent was separated from the adsorbate. After that, the solution was diluted with DI water and the Ni(II) concentration was determined through flame atomic absorption spectrophotometry (PerkinElmer, PinAAcle 900F, PerkinElmer, Inc., Waltham, MA, USA). The adsorption capacity of Ni(II) at equilibrium (q_e_, mg/g) was calculated from Equation (1):

(1) $$q_{e}=\frac{\left(C_{0}-C_{e}\right)\times V}{m}$$

where *C*_0_ (mg/L) and *C_e_* (mg/L) are the initial and equilibrium concentration, *V* (L) is the volume of the solution, and *m* (g) is the adsorbent weight.

## 3. Results and Discussion

### 3.1. Physical Properties of Wood–Zeolite Composites

**Figure 1:**
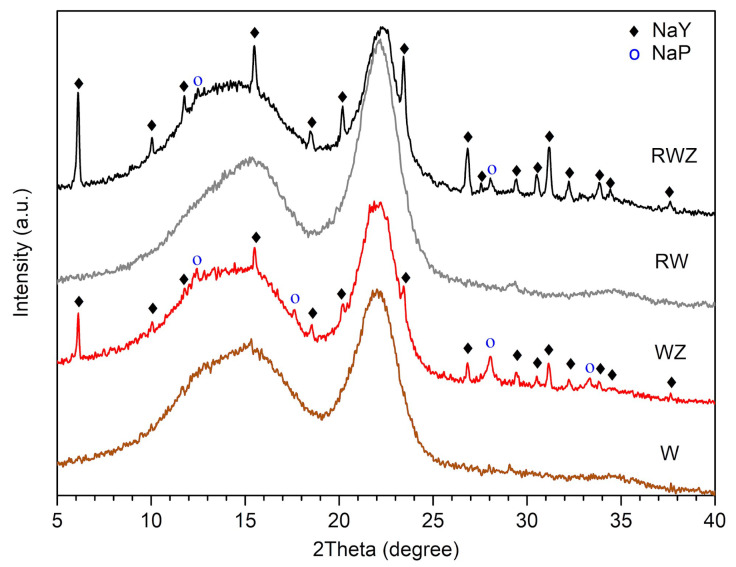
XRD patterns of wood (W), non–refluxed wood–zeolite composite (WZ), acid-refluxed wood (RW), and refluxed wood–zeolite composite (RWZ).

Figure 1 shows the XRD patterns of the non–refluxed wood (W), non–refluxed wood–zeolite composite (WZ), acid–refluxed wood (RW), and refluxed wood–zeolite composite (RWZ). All samples show broad peaks around 15° and 22°, which are characteristic of the cellulose in bamboo wood [18]. WZ and RWZ show characteristic peaks of the faujasite zeolite structure (JCPDS No. 39-1380). The peaks are more intense for RWZ, suggesting higher zeolite content. The elemental analysis revealed their Si/Al ratio of about 2 (see Table 2), confirming that the obtained zeolite is NaY. In addition, other peaks with low intensities were observed. They could be identified as zeolite NaP according to JCPDS No. 71-0962. The XRD results confirm that the synthesis of the wood–zeolite composite was successful.

The phase of zeolites could depend on the chemical structure of the wood. The non–refluxed wood contains more carboxylic acid and hydroxyl groups, as suggested by the XPS spectra of C 1s and O 1s (shown in Table S1 in the Supplementary Materials). These functional groups could trap Na^+^ ions or water molecules from the initial gel composition [19,20]. This behavior could affect the suitable gel ratio during the zeolite NaY formation and produce zeolite NaP, which requires a higher Na^+^ content [21]. This result is consistent with the previous work in which acid-refluxed lead tree wood primarily yielded zeolite NaY, whereas non–refluxed wood mainly produced zeolite NaP [11]. 

**Figure 2:**
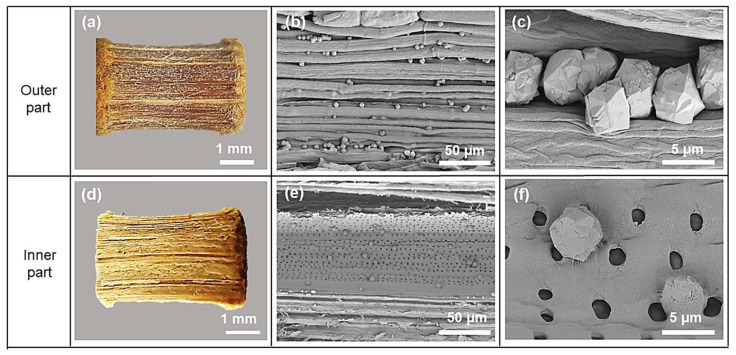
Non–refluxed wood–zeolite composite (WZ): outer part photograph (**a**) and SEM images (**b**,**c**), inner (dissected) part photograph (**d**) and SEM images (**e**,**f**).

**Figure 3:**
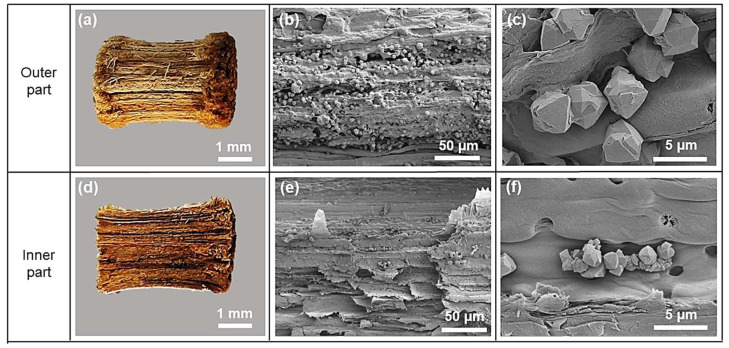
(**a**,**d**) Photographs and (**b**,**c**,**e**,**f**) SEM images of refluxed wood–zeolite composite (RWZ) from outside and inside (dissected).

Figure 2 shows photographs and SEM images of WZ. Figure 2a shows the outer part of the wood after hydrothermal treatment. The SEM image (Figure 2b) shows particles randomly distributed among the wood fibers; the majority are isolated particles. The image with a higher magnification shows that the particles are polycrystals with a diameter of about 5 μm. Figure 3d shows the inner part of the dissected wood. The SEM images (Figure 2e,f) also show zeolite crystals of a similar size randomly adhering to the walls of the phloem and xylem. The number of zeolite crystals per wood area on the outer part is significantly more than on the inner part.

Figure 3 shows photographs and SEM images of both the outer and inner parts of RWZ. The SEM image of the outer part (Figure 3b,c) shows more zeolite crystals than that of WZ. Refluxing removes hemicellulose and lignin content [22], allowing the zeolite gel to interact better with the wood. The improved interaction could contribute to a higher number of crystals. The SEM images of the inner part (Figure 3e,f) show zeolite crystals of various sizes, which are smaller than the crystals on the outer part. The number of zeolite crystals on the outer part of RWZ is also significantly higher than on the inner part. Since the zeolite gel interacts with the outer part of the wood in the first place, zeolite formation occurs more than in the inner part. 

**Figure 4:**
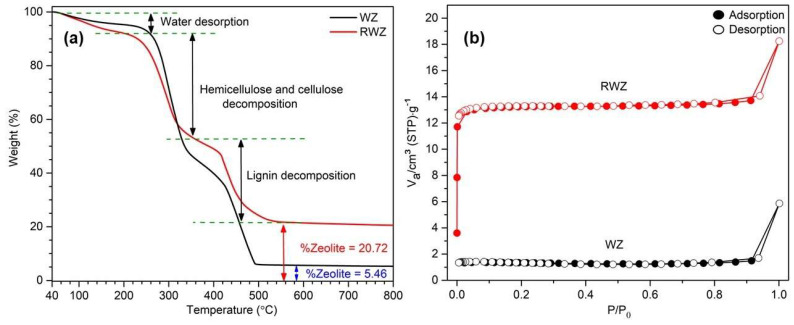
(**a**) TGA profiles and (**b**) N_2_-isotherm of untreated wood–zeolite composite (WZ) and refluxed wood–zeolite composite (RWZ).

Figure 4a displays TGA profiles of the non–refluxed wood–zeolite composite (WZ) and refluxed wood–zeolite composite (RWZ). The weight loss from both samples occurs in four temperature ranges: below 200 °C, 200–330 °C, 330–430 °C, and above 430 °C. Essentially, zeolite NaY only loses its absorbed water below 200 °C [23], while the thermal combustion of wood can be classified into three main parts consisting of hemicellulose (225–325 °C), cellulose (305–375 °C), and lignin (250–600 °C) [24]. Therefore, the calculation of zeolite content was based on the TGA results of W and WR (Figure S1 in the Supplementary Materials), which showed that all hydrocarbon compounds are completely burnt out by combustion above 800 °C [25]. Figure 4a shows that both the WZ and RWZ samples exhibit weight loss steps due to the combustion of hemicellulose and cellulose, similar to the results found in various types of wood [26]. Moreover, the functional groups of W and RW were identified through FTIR (Figure S2 in the Supplementary Materials) [27,28]. The percentage by weight of zeolite in WZ and RWZ is 5.46 and 20.72, respectively. Consistent with the FTIR results (Figure S3 in the Supplementary Materials), the relative intensities of zeolite characteristic vibrational modes in RWZ were higher than those in WZ [29]. The higher zeolite content in RWZ is consistent with previous findings by Valchev et al. [12], who suggest that higher cellulose content in the wood could lead to higher zeolite content. The cellulose content in the RW substrate is higher than in the W substrate, as determined by the thermal decomposition range of 305–375 °C and the oxidative carbon species from the XPS results (Table S2 in the Supplementary Materials) [26,30]. Furthermore, the 3D structures of a small area of WZ and RWZ from XTM suggest that the RWZ sample has a higher zeolite content, as indicated by the higher relative density area than WZ (Figure S4 in the Supplementary Materials).

Figure 4b displays the N_2_ adsorption–desorption isotherms of the composites, which exhibit two types of isotherms. The first is isotherm type I, characteristic of microporous materials from zeolite NaY [31]. The second is isotherm type II, characteristic of macroporous materials from wood [32]. Due to its higher zeolite content, RWZ exhibits a higher nitrogen uptake at low relative pressure (P/P_0_ < 0.05) than WZ. The BET surface area per gram of the WZ and RWZ composites are 5 m^2^/g and 45 m^2^/g, respectively. The increase in surface area is consistent with the zeolite content.

In summary, all characterization techniques confirm that wood–zeolite composites could be synthesized by mixing wood into NaY synthesis gel. RWZ contained significantly more zeolite NaY than WZ. The difference in zeolite phases and content would depend on the structural components of wood substrates.

### 3.2. Chemical Properties of Wood–Zeolite Composites

**Figure 5:**
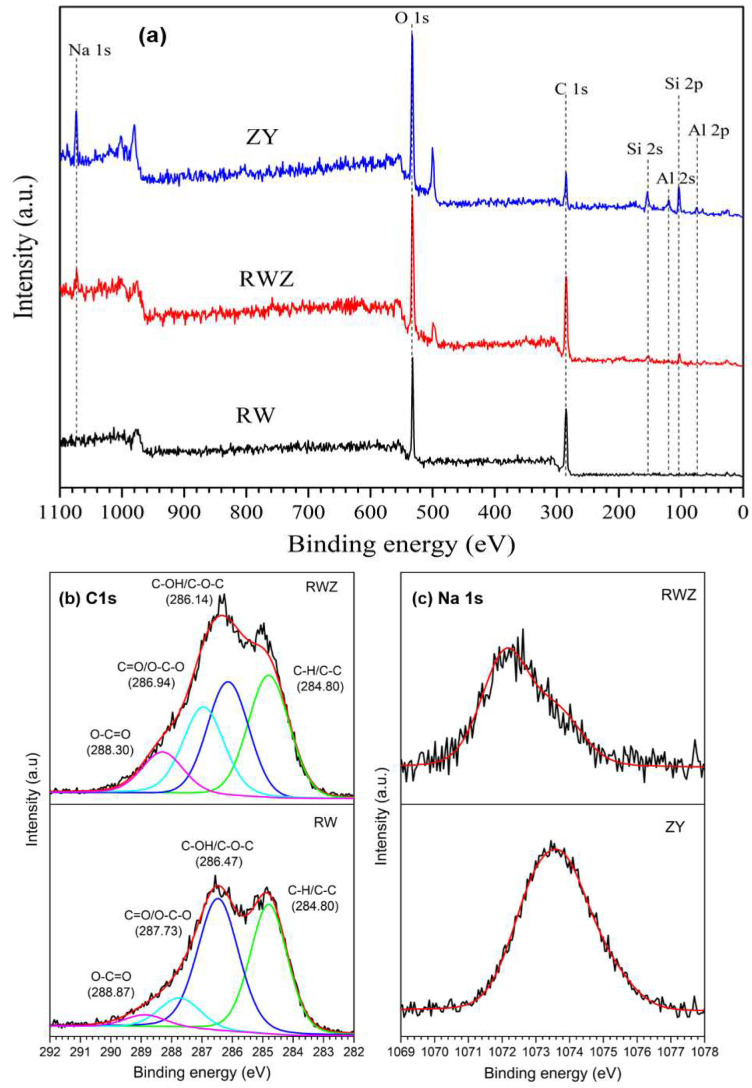
XPS spectra of full spectra (**a**), C1s (**b**), and Na 1s (**c**) of synthesized zeolite (ZY), refluxed wood (RW), and wood–zeolite composite (RWZ).

Figure 5a shows the XPS spectra in a wide–range scan of RW and RWZ compared with synthesized zeolite (ZY). ZY shows peaks corresponding to the zeolite composition, including Si and Al. The peaks from RWZ have lower intensity, while those from WZ are nearly invisible (Figure S3 in the Supplementary Materials), which is consistent with the zeolite fraction from the previous section. The chemical state of composites would be further elaborated from each binding energy. 

C 1s spectra and their relative peak–fitted area of RW and RWZ are shown in Figure 5b and Table 2. The C 1s spectra are deconvoluted into four peaks corresponding to C1_(C-C/C=C)_, C2_(H-C-O)_, C3_(H-C=O/O-C-O)_, and C4_(O=C-O)_ [33]. The oxygenated carbon (C2, C3, and C4) species in RWZ are increased, suggesting that the wood components are oxidized during wood–zeolite synthesis. This phenomenon is consistent with several studies that have reported a significant increase in the portion of oxygenated carbon in wood after treatment with alkali media [34,35]. Furthermore, the binding energy of the carboxyl group (C4_(O=C-O)_) significantly shifted to a lower value, about 0.5 eV. A few reports suggest that carboxylate anions interacted with calcium or uranium ions, driving the binding energy of C4_(O=C-O)_ to lower energy through electrostatic force [36,37]. Similar results were obtained for WZ compared to W (Figure S5 in the Supplementary Materials). The observed shift suggests that the carboxyl group of wood substrates in the composites may interact with sodium ions. The results are consistent with the Na 1s spectra (Figure 5c) containing a peak of about 1072 eV, corresponding to the R-COO-Na complex, which, together with Na, displays interaction with the zeolite framework oxygen (1073 eV) [38,39]. Figure S6 shows the O 1s spectra, which display two oxygen species separately from 529.0 to 536.5 eV in the wood [33,34] and from 529.0 to 537.0 eV in the zeolite [40]. However, the overlap makes evaluating each oxygen species in the composites difficult.

Zeolite is commonly used as a cation -exchange adsorbent due to the low Si/Al ratio, which represents the rich Na ions surrounding the zeolite surface. According to the dominant properties of wood–zeolite composites mentioned above, it would be beneficial for simple separation for metal ion removal, such as Ni(II) ions from an aqueous phase [41]. As a result, Ni adsorption in the composites is further investigated in this work. 

### 3.3. Adsorption Study

Table 3 shows the Ni(II) adsorption capacity of the wood and wood–zeolite composite samples. W and RW did not adsorb, whereas WZ adsorbed with about half of RWZ’s capacity. RWZ has a higher capacity than ZY despite the different zeolite content. The pellet might be too dense, and the inner part of the pellet might not be in contact with the solution. The adsorption on both WZ and RWZ has a high deviation because each adsorption experiment was performed with only one wood pellet, and each pellet likely has different zeolite content. The adsorption results confirm that the dispersion of zeolite NaY in refluxed wood significantly improves the adsorption of Ni(II).

The amount of Ni removed is increased by incorporating zeolite into wood compared to bare wood. The Na ions in the zeolite composites could likely exchange with Ni ions. The adsorption capacities follow the order of W < RW < WZ < ZY < RWZ, corresponding to the number of zeolites and surface areas (Table 3). This result is related to the characteristics of the wood–zeolite composite and confirms our goal of achieving high zeolite content in bamboo wood through acid treatment. Additionally, the highest adsorption capacity was observed for the RWZ composite due to the good dispersion and high internal surface area of zeolite on the treated wood, promoting the mass transfer of guest species [41]. In conclusion, the adsorption capacity increases with the amount of zeolite. 

## 4. Conclusions

Crystals of zeolite NaY were successfully dispersed onto bamboo wood by mixing the wood with zeolite gel and crystallizing through a hydrothermal method. The outer part of the wood pellets had more zeolite crystals than the inner part. The acid–refluxed wood had a significantly higher zeolite content (20.72 wt%) than the non–refluxed wood (5.46 wt%). The zeolite crystals were around 5 μm and well-dispersed on the wood surface without much agglomeration. The wood–zeolite composite was an effective adsorbent for Ni(II) ions in aqueous solutions. The adsorption capacity of the acid-treated wood composite was double that of the non–refluxed wood–zeolite composite and similar to that of a NaY pellet. The composite could be easily separated from the solution.

In terms of future perspectives, it would be worthwhile to investigate the adsorption performance of the zeolite–wood composite for other metal ions or pollutants to determine its potential for broader applications as an effective adsorbent. Furthermore, assessing the reusability and stability of the composite material over multiple adsorption–desorption cycles would be essential in evaluating its long-term effectiveness and practicality. Considering the unique properties of the zeolite–wood composite, exploring potential applications beyond adsorption, such as catalysis or separation processes, could open up new avenues for its utilization.

## Figures and Tables

**Table 1:** Examples of zeolite composites with various substrates.

Zeolite Types	Substrates	Synthesis Process	Composite Form	Application	Ref.
natural clinoptilolite	alginate	mixing clinoptilolite with alginate solution and adding CaCl_2_ to form beads	beads	adsorption	[4]
A and ZSM-5	cordierite honeycombs	dip-coating and hydrothermal	zeolite thin films	-	[5]
natural zeolite	sugarcane	powder mixing	powder	adsorption	[6]
NaX	macadamia nutshell	carbon activation and hydrothermal	powder	adsorption	[7]
NaX	rice husk	carbonization and hydrothermal	powder	adsorption	[8]
NaA	coal gangue	carbon activation and hydrothermal	powder	adsorption	[9]
NaY	cellulose filter paper	hydrothermal	filter paper	antibacterial	[10]
A	vegetal cellulose fiber sheets	immersing in gel and hydrothermal	zeolite film	-	[11]
A	vegetal cellulose fibers	immersing in gel and hydrothermal	fibers	-	[12]
NaY	lead tree wood	hydrothermal	pellet	adsorption	[13]

**Table 2:** Si/Al ratio and carbon species of WZ and RWZ wood–zeolite composites.

Sample	Si/Al Ratio from EDS *	Deconvoluted Peak Area of XPS C 1s (%)			
		C1_(C-C/C=C)_	C2_(H-C-O)_	C3_(H-C=O/O-C-O)_	C4_(O=C-O)_
WZ	2.08 ± 0.06	20.67	15.25	46.83	17.25
RWZ	2.13 ± 0.13	33.30	30.04	24.93	11.73

* Average values from 9 areas on the zeolite particles, determined through SEM-EDS (see Figure S7).

**Table 3:** Ni(II) adsorption capacity of wood, wood–zeolite composites, and NaY.

Sample	Adsorption Capacity, q_e_ (mg_Ni(II)/_g_adsorbent_) *
Bare wood (W)	Not adsorbed
Refluxed wood (RW)	Not adsorbed
Wood–zeolite (WZ)	4.90 ± 1.22
Refluxed wood–zeolite (RWZ)	9.51 ± 1.79
NaY (ZY)	8.63 ± 0.88

* Average value ± standard deviation, n = 3.

## Data Availability

Not applicable.

## Supplementary Materials

The following supporting information can be downloaded at: https://www.mdpi.com/article/10.3390/ma16144946/s1, Table S1: Peak-fitted C 1s and O 1s from XPS data.; Table S2: Thermal degradation of non–refluxed wood (W) and refluxed wood (RW) in zero air flow.; Figure S1: TGA profile and C 1s spectra of W and RW.; Figure S2: FTIR spectra of W and RW.; Figure S3: FTIR spectra of WZ, RWZ, and ZY.; Figure S4: 3D X-ray tomography images of non–refluxed wood–zeolite composite (WZ) and refluxed wood–zeolite composite (RWZ).; Figure S5: XPS spectra of C 1s of non–refluxed wood (W) and wood–zeolite composite (WZ).; Figure S6: O 1s, Al 2p, and Si 2p spectra of WZ, RWZ, and ZY.; Figure S7: SEM images and EDS spectra of WZ and RWZ samples.
