# Supplementary material for: Synthesis and Characterization of Zeolite NaY Dispersed on Bamboo Wood
Source: Materials (Basel). 2023 Jul 11;16(14):4946. doi: 10.3390/ma16144946 (PMC10381651; doi:10.3390/ma16144946)
Supplement: Supplementary file 1 [file materials-16-04946-s001.zip › materials-2459130-supplementary.docx]

Supplementary Materials

Synthesis and Characterization of Zeolite NaY Dispersed on Bamboo Wood

Pimrapus Tawachkultanadilok, Nattawut Osakoo, Chalermpan Keawkumay, Krittanun Deekamwong, Narongrit Sosa, Catleya Rojviriya, Supinya Nijpanich, Narong Chanlek, Sanchai Prayoonpokarach * and Jatuporn Wittayakun *

**Table S1.** Peak-fitted C1s and O1s from XPS data of non-refluxed wood.

| **Samples** | **C1s** | | | | **O1s** | | |
| --- | --- | --- | --- | --- | --- | --- | --- |
|  | **C1**  **_(C*-C/C*=C)_** | **C2**  **_(H-C*-O)_** | **C3**  **_(H-C*=O/O-C*-O)_** | **C4**  **_(O=C*-O)_** | **C=O/ O-C=O** | **C-O** | **C=O** |
| W | 49.91 | 30.80 | 11.68 | 7.61 | 19.18 | 59.38 | 21.44 |
| RW | 40.49 | 45.20 | 10.10 | 4.21 | 9.01 | 81.83 | 9.16 |

**Table S2.** Thermal degradation of non-refluxed wood (W) and acid-refluxed wood (RW) in air zero flow.

| **Samples** | **Water evaporation** | | **1^st^ step** | | **2^nd^ step** | | **3^rd^ step** | |
| --- | --- | --- | --- | --- | --- | --- | --- | --- |
|  | **wt. lost  [%]** | **T_d_  [°C]** | **wt. lost [%]** | **T_d_ [°C]** | **wt. lost [%]** | **T_d_ [°C]** | **wt. lost [%]** | **T_d_ [°C]** |
| W | 3 | 71 | 7 | 195 | 56 | 287 | 34 | 422 |
| RW | 3 | 70 | - | - | 71 | 320 | 26 | 502 |


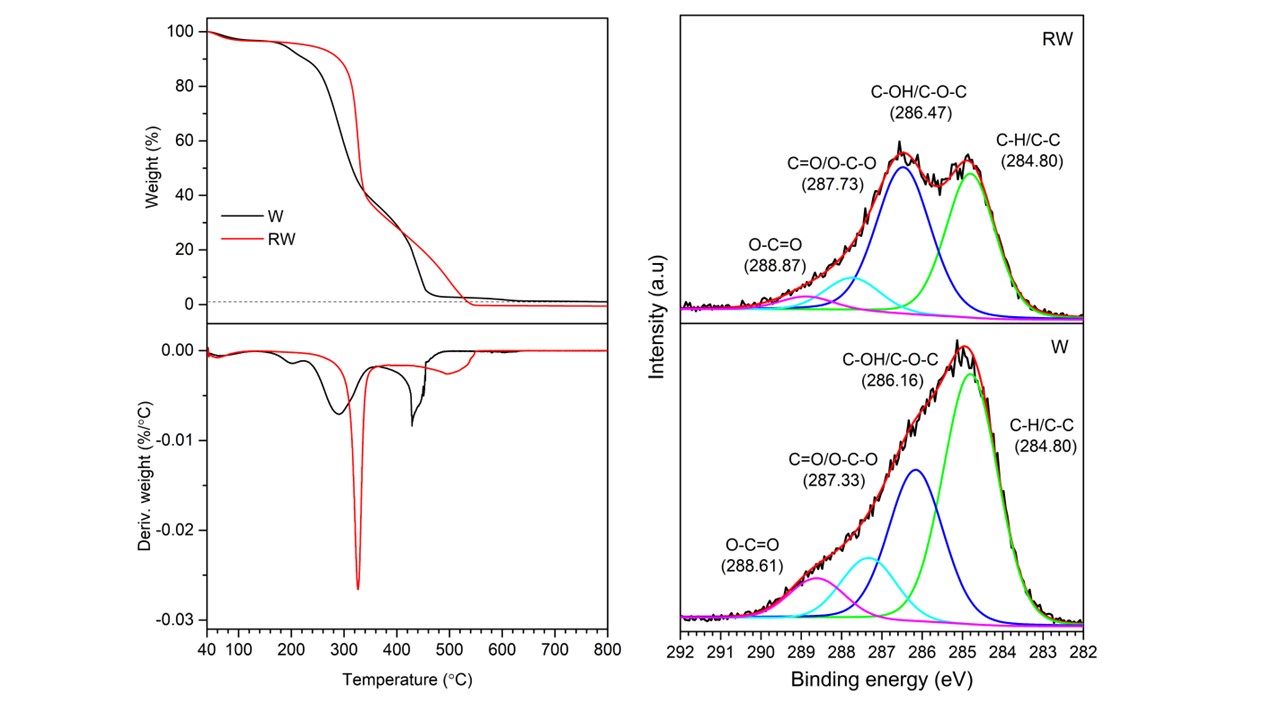


**Figure S1.** TGA profile and C 1s spectra of W and RW.


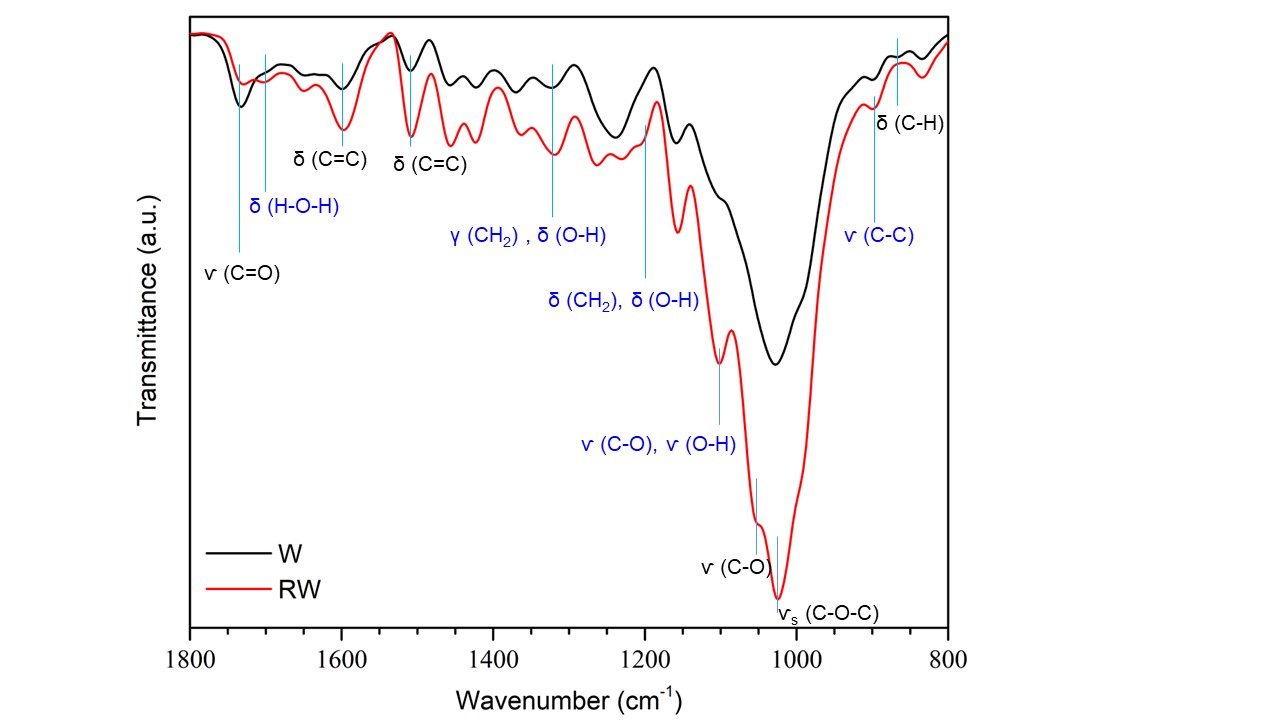


**Figure S2.** FTIR spectra of W and RW.


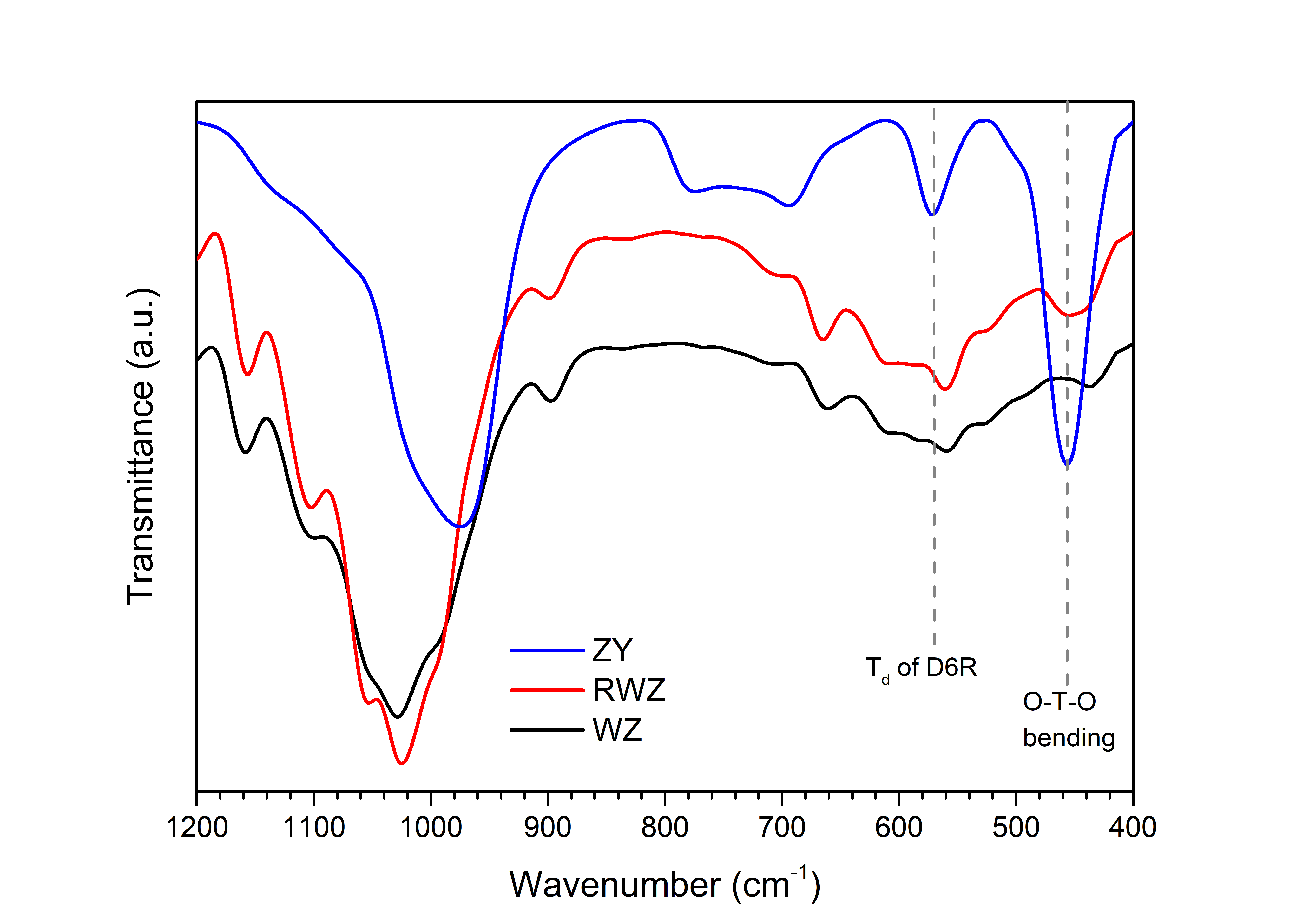


**Figure S3.** FTIR spectra of WZ, RWZ, and ZY.


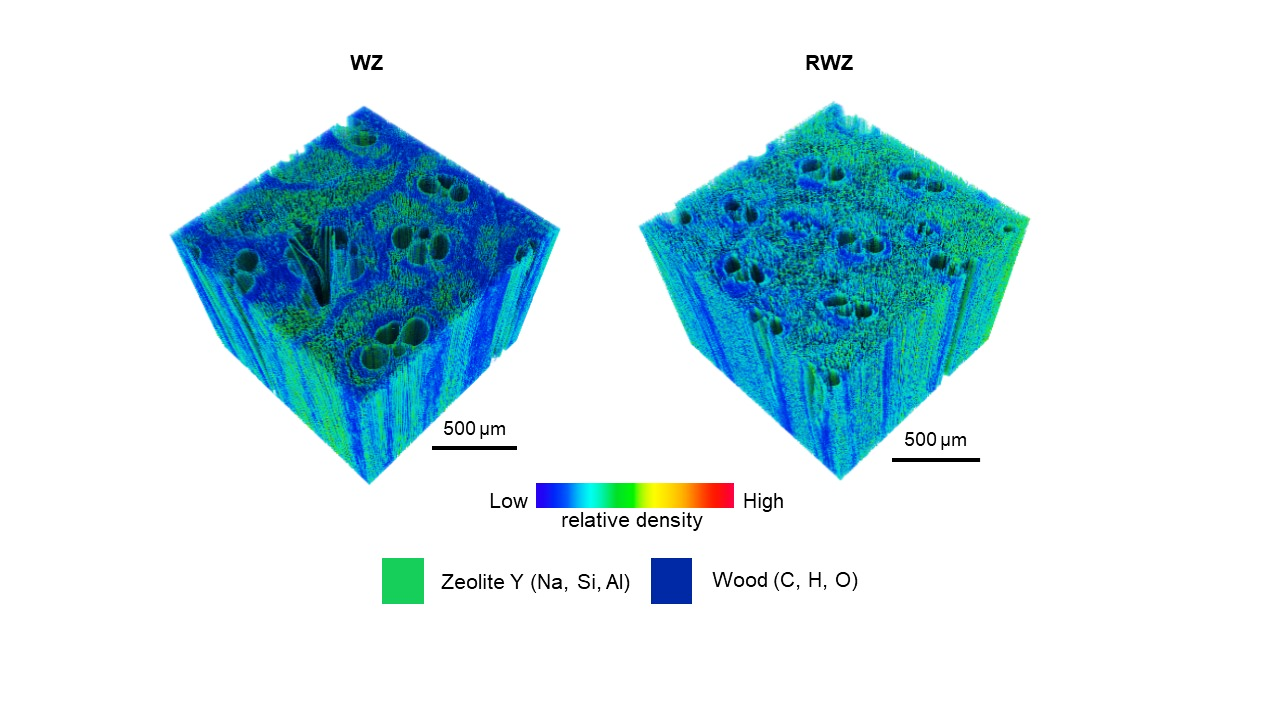


**Figure S4.** 3D X-ray tomography images of untreated wood-zeolite composite (WZ) and refluxed wood-zeolite composite (RWZ).


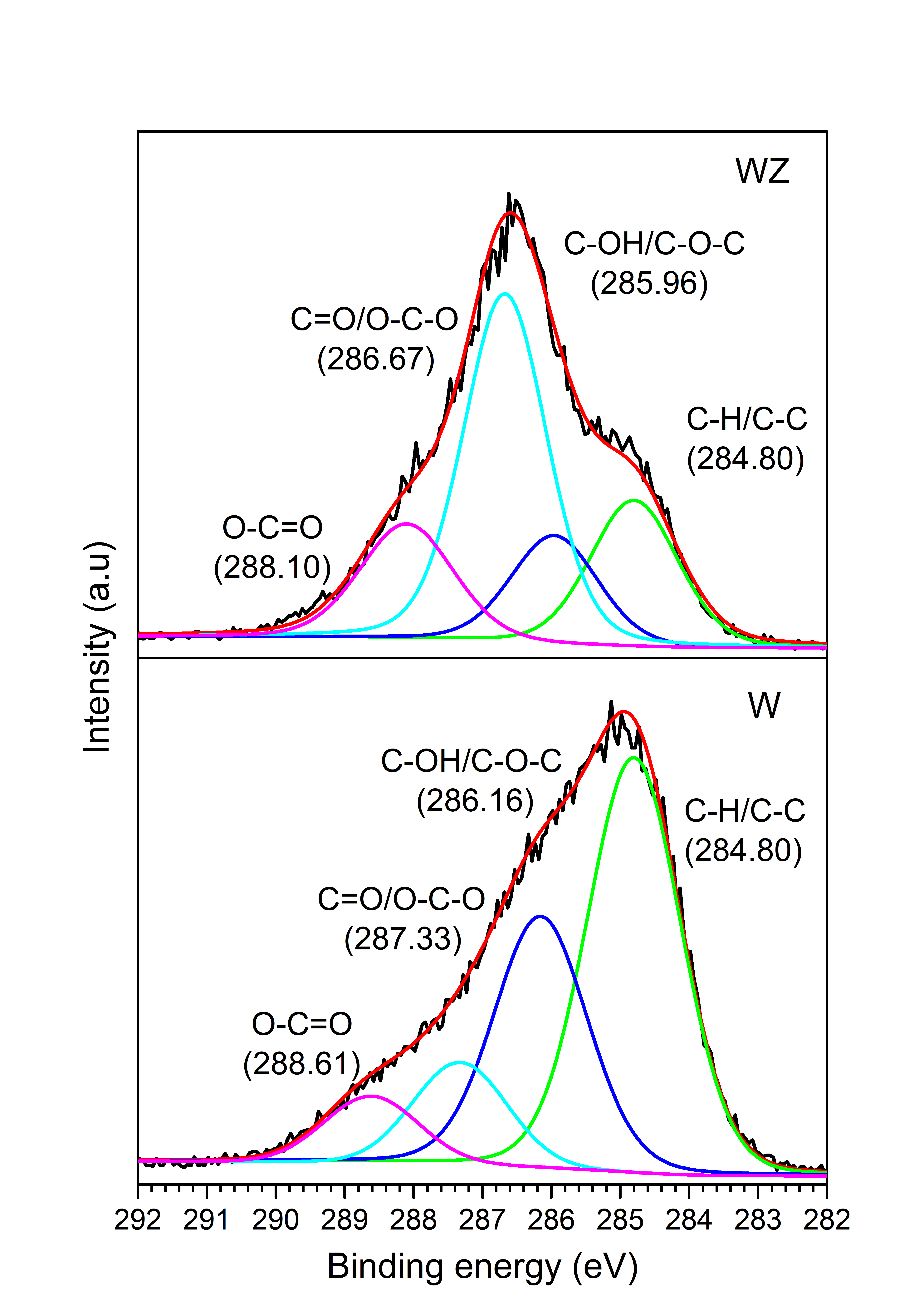


**Figure S5.** XPS spectra of C 1s of untreated wood (W) and wood-zeolite composite (WZ).
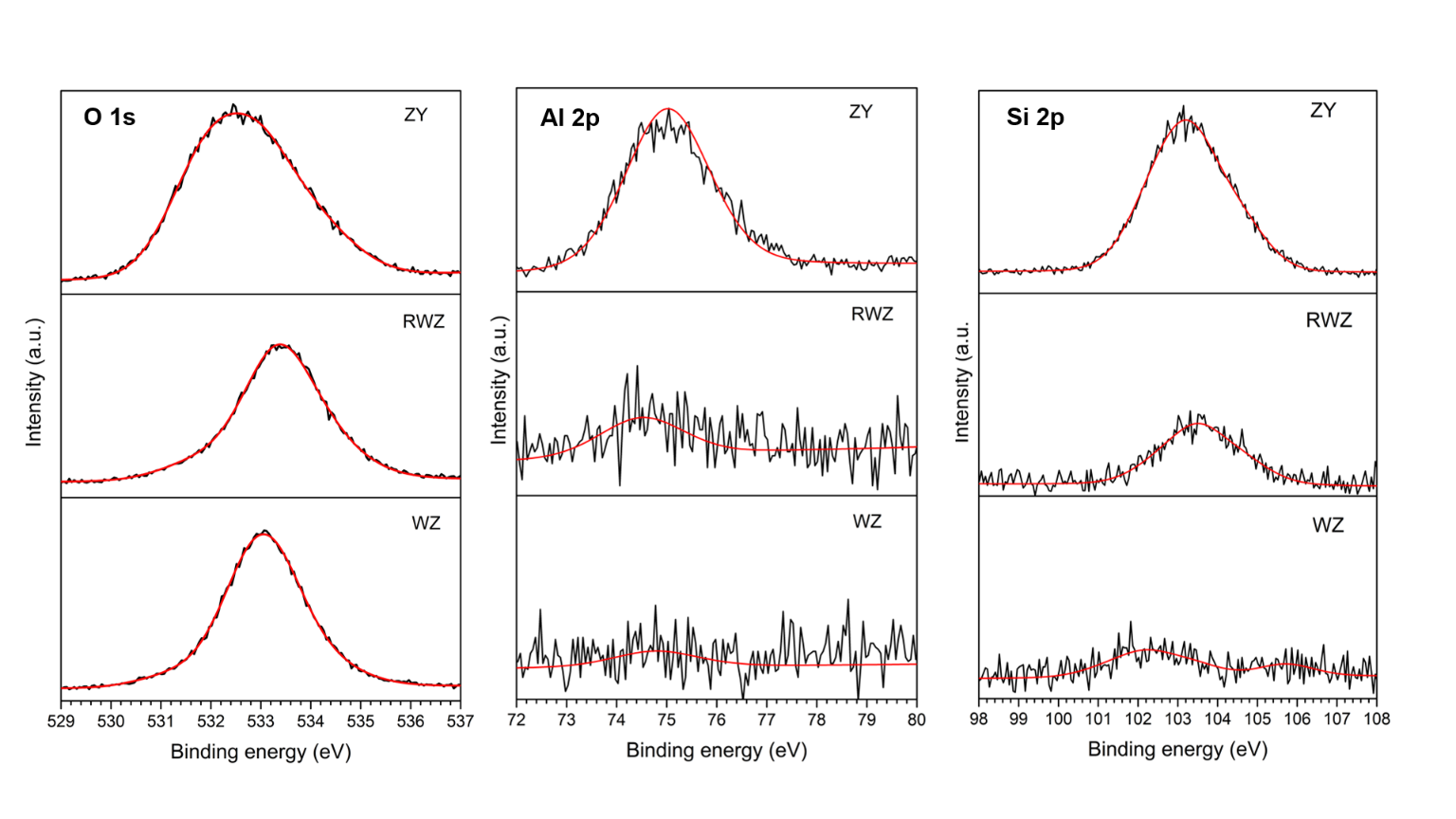


**Figure S6.** O 1s, Al 2p and Si 2p spectra of WZ, RWZ, and ZY.


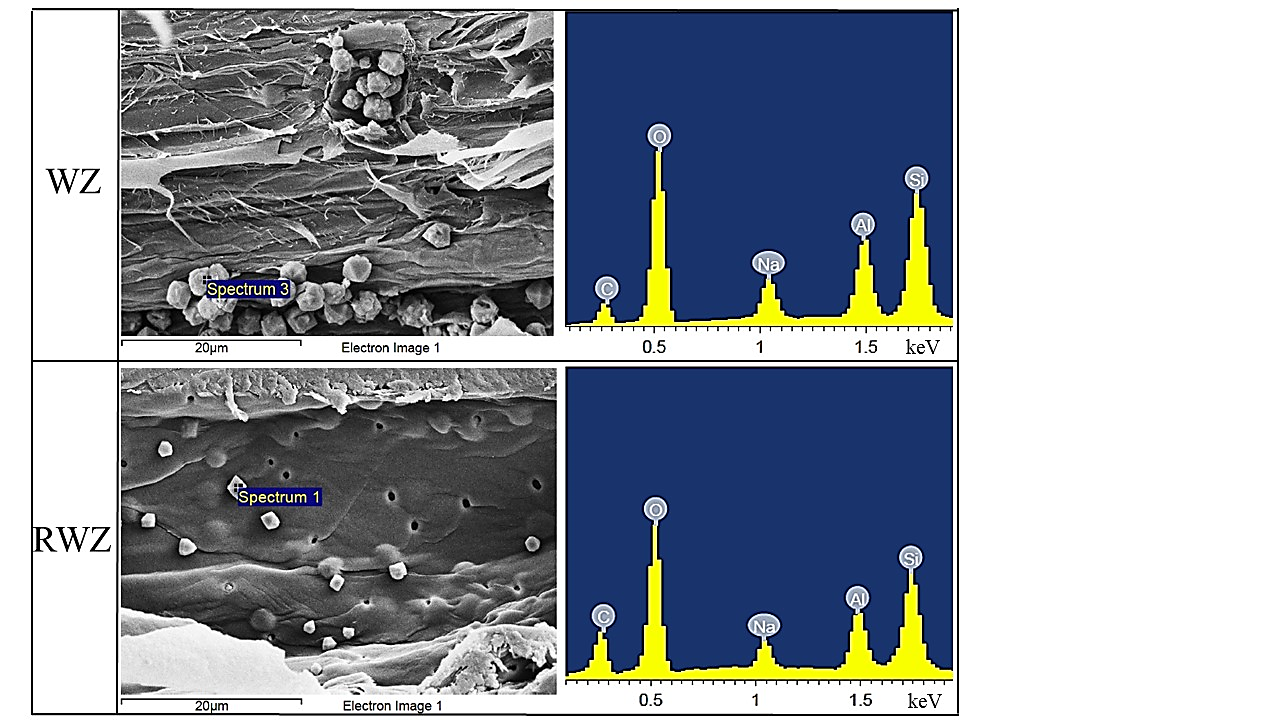


**Figure S7.** SEM images and EDS spectra of WZ and RWZ samples.
